# Pre‐operative templating for total hip arthroplasty: How does radiographic technique and calibration marker placement affect image magnification?

**DOI:** 10.1002/jmrs.461

**Published:** 2021-02-15

**Authors:** Mia Holliday, Adam Steward

**Affiliations:** ^1^ Western Health Footscray Victoria Australia; ^2^ Deakin University Geelong Victoria Australia

**Keywords:** Digital radiography, magnification, pre‐operative planning, scaling marker, total hip arthroplasty

## Abstract

**Introduction:**

Pre‐operative templating using digital radiography is an effective method of planning for total hip arthroplasty (THA) and requires a generalised fixed magnification factor (MF) or external calibration markers (ECM). The effect on image magnification when changing source‐to‐image distance (SID), object‐to‐image distance (OID) and different imaging conditions is not well described. This study aims to quantify the range of effects manipulation of radiographic parameters can have on image magnification across different body habitus and imaging conditions.

**Methods:**

A simple phantom study was performed. A 25 mm ECM was placed at eight different OID values along the anterior–posterior phantom plane at three different SID values and imaging conditions, and X‐rays were obtained. On each radiograph, the ECM was measured using a line calliper tool by three radiographers and recorded. The MF was calculated and recorded.

**Results:**

The smallest observed image MF was 1.16, for an 8 cm OID, 120 cm SID with the ECM placed within the central ray and the X‐ray detector in bucky underneath the X‐ray table. The largest image MF was 1.40 for a 15 cm OID, 100 cm SID with the X‐ray detector placed underneath an emergency department imaging trolley.

**Conclusions:**

Digital pre‐operative templating for THA relies on accurate radiographic positioning and is dependent of the patient body habitus, radiographic parameters and imaging conditions selected by the radiographer. The use of appropriately positioned ECMs – placed medially between the patient’s internally rotated legs at the level of the greater trochanter, lowers the potential for magnification inaccuracies.

## Graphical abstract

Digital preoperative templating for total hip arthroplasty (THA) relies on accurate radiographic positioning, and is dependent of the patient body habitus, radiographic parameters and imaging conditions selected by the performing radiographer. Based on the findings of this study, we believe that the use of a generalised magnification factor (MF) does not account for deviation of these factors, and hence can be inaccurate. The use of appropriately positioned external calibration markers (ECM) – placed medially between the patient’s internally rotated legs at the level of the greater trochanter, lowers the potential for magnification inaccuracies.

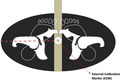

## Introduction

Total hip arthroplasty (THA) has become a well‐established and successful interventional procedure for the treatment of hip pain and poor joint function.^1^, ^2^, ^3^ Pre‐operative templating using digital radiography is an effective method of planning for THA.^2^, ^4^, ^5^, ^6^, ^7^, ^8^, ^9^, ^10^ This method allows for the surgeon to estimate the size of a component from the size of a small metal ball known as an external calibration marker (ECM), or through the use of a fixed magnification factor (MF) (See https://www.materialise.com/en/medical/orthoview/smart‐planning for further information). However, these methods rely on accurate and consistent radiographic positioning by the radiographer, which anecdotally can be poorly executed in clinical practice. To our knowledge, no significant research has been conducted to determine the variance of effects inconsistent radiographic positioning has on image magnification. This study aims to quantify the range of effects manipulation of radiographic parameters can have on image magnification, and how this can be applied across different patient body habitus and imaging conditions.

**Figure 1:**
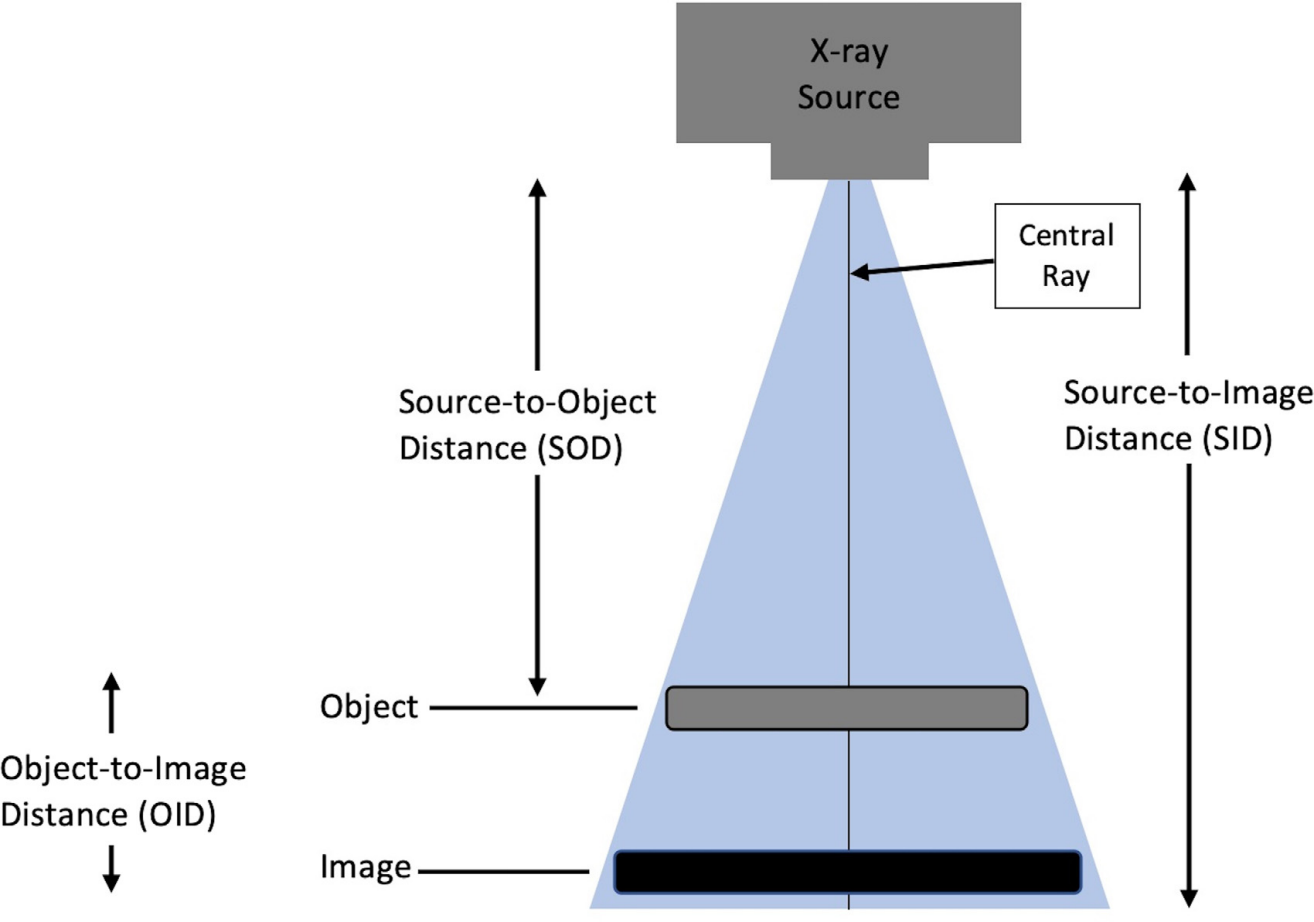
The Magnification Factor (MF) of a radiographic image is the ratio between source‐to‐image receptor distance (SID) and source‐to‐object distance (SOD).

The primary goal of total hip arthroplasty (THA) is pain reduction and restoration of normal hip function, whilst limiting the rate of intra‐operative and post‐operative complications.^3^, ^10^, ^11^, ^12^ This can be achieved via accurate pre‐operative radiographic pelvic templating prior to surgical intervention. Pelvic templating relies on an understanding of the theory behind divergence of ionising radiation from a point source, and more specifically the factors, which can affect the magnification of a radiographic image: source‐to‐image distance (SID), source‐to‐object distance (SOD) and lateral positioning (See Fig. 1).^13^, ^14^, ^15^, ^16^

The value of radiographic pre‐operative templating for THA is well described in the literature. Digital templating is the current gold‐standard method of pre‐operative templating for THA compared with traditional acetate templating techniques.^4^, ^5^, ^7^ Digital templating utilises specialised imaging software to calibrate true distances along a digital radiograph using an external calibration marker (ECM) or fixed magnification factor (MF). There is still debate as to which digital templating technique is most reliable and accurate in clinical practice. Some clinical centres prefer the use of a fixed MF,^6^, ^10^, ^12^, ^17^, ^18^ as it can provide consistent templating results that are less dependent on radiographic positioning. This method was also used in traditional acetate templating, with a typical assumed MF of 110 to 120%.^10^, ^15^, ^19^, ^20^ Comparatively, many centres are much more reliant on the use of ECMs, which are externally placed at the level of a patient’s hip joint by the radiographer.^1^, ^11^, ^13^ The vast majority of studies that discussed templating using an ECM noted mild to significant inaccuracies most likely due to inaccurate ECM placement from the radiographer.^1^, ^10^, ^11^, ^13^, ^15^, ^21^ Several studies went on to hypothesise that these inaccuracies may be due to poor understanding and consistency of radiographic parameters which can affect magnification. These parameters include SOD, SID and lateral placement of an ECM.^13^, ^14^, ^15^, ^20^

Accurate and reliable pre‐operative templating is largely dependent upon the skill and consistency of the radiographer and knowledge of geometric magnification parameters. It can be difficult to locate the centre of the hip joint, hence in clinical practice the greater trochanter is used as a radiographic landmark for radiographers to determine the appropriate height for ECM placement. This height known as the object‐to‐image distance (OID), can be used to calculate the SOD of an image, and hence the degree of image magnification.^16^ Therefore, although it has been hypothesised within the literature,^14^, ^20^, ^21^ it does not appear that there is any quantitative data on the effects varying SOD distance has on image magnification. Furthermore, it has also been hypothesised that patient body habitus and body mass index (BMI) could have an effect on positioning accuracy due to its potential relationship to SOD,^3^, ^9^, ^15^, ^18^, ^21^ albeit no significant quantitative data has been conducted within the literature thus far.

SID is a known radiographic parameter that influences image magnification.^14^, ^20^ Yet within clinical practice, manipulation of SID does not appear to be a widely explored nor controlled radiographic technique. The investigated literature provided a wide variation of SID values for pelvic templating, between 100 and 120 cm.^5^, ^6^, ^11^, ^18^, ^20^, ^22^ However, no studies to our knowledge have specifically investigated the effects SID has on image magnification. Lastly, is also important to consider how these vertical parameters are influenced by lateral placement of an ECM. There are currently two lateral positioning methods implemented in clinical practice. It is generally accepted that when an ECM is placed medially between the patient’s legs at the level of greater trochanter, there is a smaller effect on image magnification.^7^, ^11^, ^13^ However, one can also place the ECM laterally, which is anecdotally much more comfortable for the patient and radiographer and the introduced error is believed to be clinically insignificant.^9^, ^13^, ^18^

The primary objective of this study was to determine if there are significant clinical effects on image magnification depending on greater trochanter height (OID) and manipulation of SID and lateral ECM placement. This has been hypothesised within the literature; however, no significant conclusions have been reached to our knowledge. Furthermore, our aim was to determine the image magnification variance when changing SID and lateral marker placement for different OID values. It is well documented within the literature that a precision of one component size between the templated and implanted prosthesis components is clinically acceptable.^1^, ^4^, ^5^, ^7^, ^10^, ^11^, ^23^ It is our hypothesis that the clinical decisions made by the radiographer will influence the precision of the templating technique.

## Materials and Methods

A simple phantom study was performed. Ethics approval was not required, nor sought.

Prior to the commencement of this study, table‐to‐trochanter distance was measured using the author’s own body habitus and extrapolated to a range that would anecdotally fit the local population. The smallest extrapolated table‐to‐trochanter distance (OID) was 8 cm. The largest was 15 cm. This should fit most population sizes; however, it must be noted that as a non‐clinical collection of data, smaller and larger distances may be represented in clinical practice.

**Figure 2:**
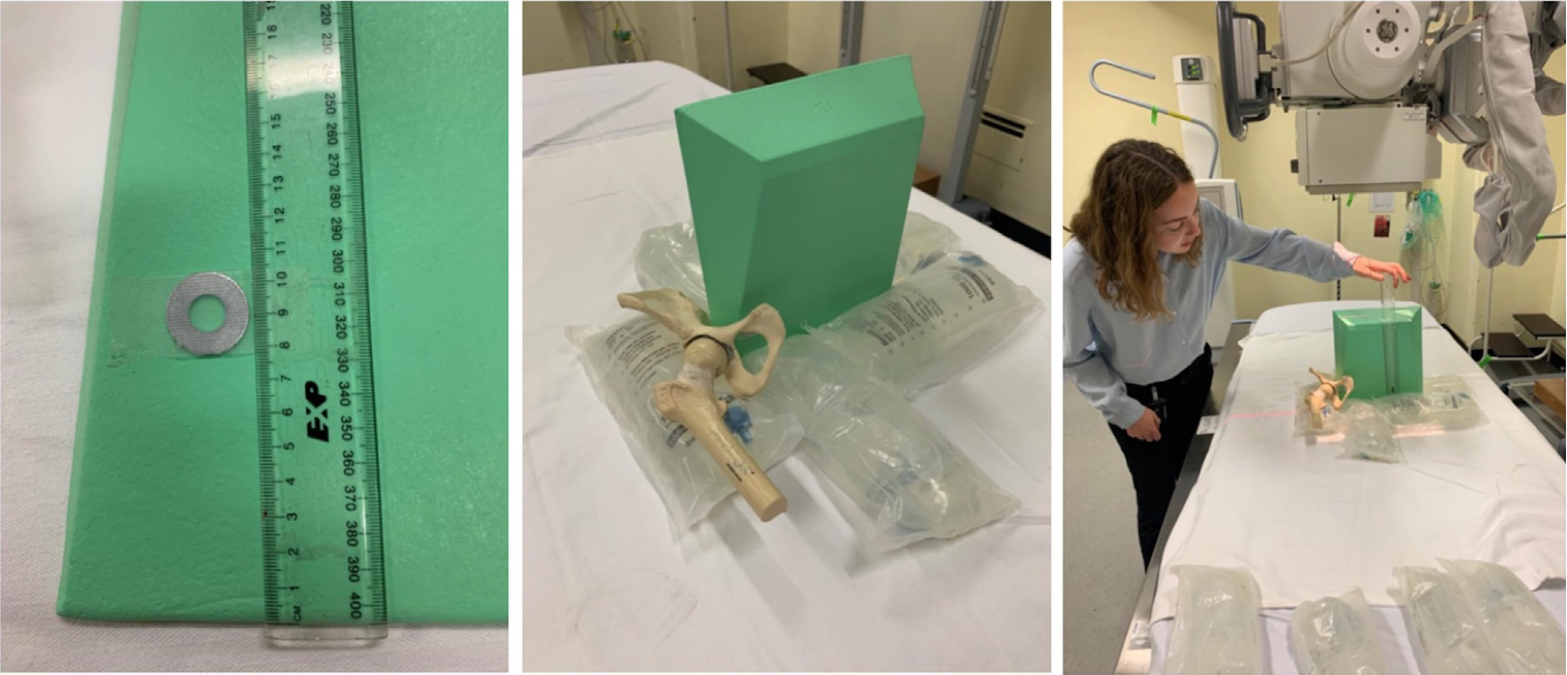
X‐ray phantom set‐up.

A 25 mm external calibration templating marker disc was used for this study. It was taped onto a radiolucent sponge at measured distances from the radiographic tabletop and positioned vertically on a traditional X‐ray table as shown in Figure 2. This aimed to mimic the normal X‐ray set‐up for a supine AP pelvis X‐ray.

An initial radiograph was taken using this set‐up with the marker disc placed 8 cm from the table‐top adjacent to the phantom’s hip centre. A 100 cm FFD was used. Additional radiographs were taken with the marker disc placed at OIDs of 9, 10, 11, 12, 13, 14 and 15 cm from the X‐ray table. The same conditions were then applied to a 110 cm and 120 cm FFD, respectively and X‐rays were taken at the aforementioned OID values. With the ECM placed to the lateral edge of the X‐ray field at each previously mentioned OID value, images were obtained at 100, 110 and 120 cm FFD. These SID values were chosen as they were most commonly observed within the literature and most reflective of clinical practice. Finally, the same trial was completed with the X‐ray detector placed underneath an ED trolley, for each OID value at 100, 110 and 120 cm FFD with the ECM placed medially within the CR. This mimics current practice to image the trauma pelvis under the patient trolley, observed in emergency department X‐ray, as it reduces transfer of the patient to the radiographic table (See Figure S1).

All radiographs were taken using a GE 656 Discovery Direct Digital Radiography Unit with ‘Flash‐Pad’ flat panel detectors (GE Healthcare, Chicago, USA). The X‐ray machine is regularly serviced and maintained by the GE service team. No formal quality control checks were performed outside the regular quality control checks during servicing on the measurement tools, which exposes a potential limitation to the study. The radiographs were measured using the GE line calliper tool. Three radiographers measured the imaged size of the templating marker disc on each radiograph using the line calliper tool (See Figure S2). Prior to the formal measurements, each radiographer underwent trial measurements to attempt to maintain consistency across the three radiographers. Each radiographer performed only one set of measurements introducing the potential for intra‐observer error. An average of the three trials was then used to calculate the average measurement of the image. The magnification factor (MF) was calculated using the following equation:

$$\text{Magnification}\;\text{Factor}\;\left(\text{MF}\right)=\text{Image}\;\text{Size}/\text{Object}\;\text{Size}$$

As another means of ensuring and assessing accuracy for our predicted measurements, the expected magnification for each value was mathematically derived using similar triangles to obtain the expected image size of the ECM. The procedure for this is provided in the Supporting Information (See Figure S3) and the mathematical measurements are tabled in comparison with measured ECM sizes (See Tables S1–S3).

## Results

The results are shown in Tables 1, 2, 3, 4. Tables 1, 2, 3 show the average measured disc sizes between three radiographers and the calculated MF at 100, 110 and 120 cm SID values for the three imaging conditions. Tables S1–S3 in Supporting Information show the measured values for each observer. As shown in Table 4, there is large variation in the calculated MF between different X‐ray position set‐ups. Most notably, the smallest calculated MF with medial ECM placement is 1.16 when the templating disc is placed at an OID of 8 cm within the central ray at 120 cm SID with the detector in bucky under the X‐ray table. Comparatively the largest calculated MF is 1.40 at 15 cm OID, with the detector placed under the emergency department trolley at 100 cm SID. This accounts for a difference of 0.24 in image magnification.

**Table 1:** Average disc measurements at 100, 110 and 120 cm SID values.

OID (cm)	ECM Measured Average 100 cm SID (mm)	MF 100 cm	ECM Measured Average 110 cm SID (mm)	MF 110 cm	ECM Measured Average 120 cm SID (mm)	MF 120 cm
8	29.7	1.19	29.6	1.18	28.9	1.16
9	30.0	1.20	29.8	1.19	29.3	1.17
10	30.3	1.21	30.1	1.20	29.6	1.19
11	31.1	1.24	30.5	1.22	29.8	1.19
12	31.6	1.26	30.7	1.23	30.2	1.21
13	31.7	1.27	31.1	1.24	30.4	1.22
14	32.3	1.29	31.6	1.27	30.4	1.22
15	32.9	1.31	31.9	1.28	31.0	1.24

External calibration marker (ECM) placed with the central ray, X‐ray detector placed in bucky underneath the X‐ray table.

OID, object‐to‐image distance; MF, magnification factor; SID, source‐to‐image distance.

**Table 2:** Average disc measurements at 100, 110 and 120 cm SID values.

OID (cm)	ECM Measured Average 100 cm SID (mm)	MF 100 cm	ECM Measured Average 110 cm SID (mm)	MF 110 cm	ECM Measured Average 120 cm SID (mm)	MF 120 cm
8	29.8	1.19	29.5	1.18	28.9	1.15
9	30.2	1.21	29.7	1.19	29.1	1.16
10	30.6	1.23	30.0	1.20	29.2	1.17
11	31.1	1.24	30.4	1.21	29.8	1.19
12	31.4	1.25	30.6	1.22	30.0	1.20
13	31.5	1.26	31.3	1.25	30.5	1.22
14	32.0	1.28	31.4	1.25	30.9	1.23
15	32.4	1.30	31.7	1.27	31.2	1.25

External calibration marker (ECM) placed on the lateral edge of the detector, X‐ray detector placed in bucky underneath the X‐ray table.

OID, object‐to‐image distance; MF, magnification factor; SID, source‐to‐image distance.

**Table 3:** Average disc measurements at 100, 110 and 120 cm SID values.

OID (cm)	ECM Measured Average 100 cm SID (mm)	MF 100 cm	ECM Measured Average 110 cm SID (mm)	MF 110 cm	ECM Measured Average 120 cm SID (mm)	MF 120 cm
8	32.0	1.28	31.3	1.25	31.1	1.25
9	32.8	1.31	31.7	1.27	31.6	1.26
10	33.2	1.33	32.1	1.28	31.9	1.28
11	33.2	1.33	32.8	1.31	32.2	1.29
12	34.0	1.36	33.2	1.33	32.5	1.30
13	34.3	1.37	33.3	1.33	33.0	1.32
14	34.6	1.39	34.0	1.36	33.1	1.33
15	35.0	1.40	34.1	1.37	33.3	1.33

External calibration marker (ECM) placed within the central ray, X‐ray detector placed underneath the ED trolley.

OID, object‐to‐image distance; MF, magnification factor; SID, source‐to‐image distance.

**Table 4:** The magnification factors (MF) of the imaged OID values at 100, 110 and 120 cm SID values for three chosen imaging conditions.

OID (cm)	MF Table 100 cm	MF Table 110 cm	MF Table 120 cm	MF Lateral 100 cm	MF Lateral 110 cm	MF Lateral 120 cm	MF Trolley 100 cm	MF Trolley 110 cm	MF Trolley 120 cm
8	1.19	1.18	**1.16**	1.19	1.18	1.15	1.28	1.25	1.25
9	1.20	1.19	1.17	1.21	1.19	1.16	1.31	1.27	1.26
10	1.21	1.20	1.19	1.23	1.20	1.17	1.33	1.28	1.28
11	1.24	1.22	1.19	1.24	1.21	1.19	1.33	1.31	1.29
12	1.26	1.23	1.21	1.25	1.22	1.20	1.36	1.33	1.30
13	1.27	1.24	1.22	1.26	1.25	1.22	1.37	1.33	1.32
14	1.29	1.27	1.22	1.28	1.25	1.23	1.39	1.36	1.33
15	1.31	1.28	1.24	1.30	1.27	1.25	**1.40**	1.37	1.33

Table: ECM within the central ray, X‐ray detector in bucky underneath the X‐ray table. Lateral: ECM on the lateral edge of the detector, X‐ray detector placed in bucky underneath the X‐ray table. Trolley: ECM placed within the central ray; X‐ray detector placed underneath the ED trolley.

OID, object‐to‐image distance; MF, magnification factor. The two figures in bold represent the two extremes of magnification (the greatest and the least).

Mathematically calculated measurements varied by no greater than 1.7 mm for the images performed in the table bucky and by no more than 1.2 mm for those performed under the ED trolley. These results are tabulated in Tables S1–S3.

## Discussion

As shown in Table 4, there was large variation in the calculated MF between different X‐ray position set‐ups. The smallest calculated MF with medial ECM placement is 1.16 (116%) when the templating disc was placed at an OID of 8 cm within the central ray at 120 cm SID with the detector in bucky under the X‐ray table. The largest calculated MF is 1.40 (140%) at 15 cm OID, with the detector placed under the emergency department trolley at 100 cm SID.

This is important in the context of using fixed magnification, which assumes a typical fixed MF of 110 to 120%.^10^, ^15^, ^19^, ^20^ This highlights the potential inaccuracies with fixed magnification as it is largely dependent on positioning parameters. Within the literature there is a preference for the use of fixed MF.^6^, ^10^, ^12^, ^17^, ^18^, ^19^ However our results suggest that fixed MF values of 110–120% could be too low for accurate templating. To our knowledge, studies that prefer the use of a fixed MF have neglected to mention the effect of patient body habitus. From our results, it is only when a small OID of less than 12 cm representing a ‘small to average’ patient body habitus that a MF of less than 120% can be achieved (See Table 4). Furthermore, in trauma imaging, where ED trolleys are increasingly being used as an imaging option, less than 120% of magnification cannot be achieved for our smallest OID value of 8 cm. In order to minimise this potential error from imaging conditions (SID and imaging apparatus) and patient body habitus (OID), we suggest that the use of correctly positioned ECMs is more accurate.

It must be noted that a study performed by Hornová et al.^12^ did take patient body habitus into consideration. Despite identical radiological set‐up at each site and a homogenous patient cohort, they found that MF was dependent upon the clinical site it was performed and not on patient size. They concluded that to improve the accuracy of using fixed magnification, it is not accurate to use generalised MF values rather the optimal value of fixed MF should be calculated for each individual clinical site. These results identify the discrepancy that exists across clinical sites and variation that occurs due to radiographer technique. It supports the clinical impact that the results of our study bring to light.

Kniesel et al.^21^ found that the correlation between pre‐operatively templated and implanted component size improved with the use of an ECM, however, was weak overall. They compared the use of an ECM with a fixed MF of 110%. Without the use of an ECM there was a tendency for the predicted component size to be too large. Our results confirm this (See Table 4). Archibeck et al.^10^ compared ECM and 121% fixed magnification templating techniques. They concluded that a fixed magnification is clinically less time consuming and less dependent on positioning error. As this was a prospective study, it is likely that the radiographic technique was much more tightly regulated than in the previous studies, which may explain their higher amount of accurately predicted component sizes. It was also noted by the authors that a single employee with over 20 years of experience within the orthopaedic clinic performed all radiographic templating. From our results it is also evident that a fixed MF of 121% is likely much more accurate than the typically used 110–120% described within the literature, as it is much more representative of the potential variation in patient sizes. However, it still does not account for the variation in positioning techniques demonstrated in our results that is much more typical for many radiographers in clinical practice.

Furthermore, Boese et al.^8^ and Sinclair et al.^11^ found that the use of an ECM did not greatly improve pre‐operative templating techniques. Specifically, Sinclair et al.^11^ calculated that there was a percentage difference comparable to a potential inaccuracy of 6 mm, representing up to three component sizes. However, both articles noted the accuracy of this templating method is largely dependent upon the radiographer, and as retrospective analyses were not able to assess the positioning techniques used.

Within the literature, many studies acknowledged the effect of SOD and patient body size,^8^, ^14^, ^19^ however, did not explore the clinical significance this can have on component sizing. Ramme et al.^15^ attempted to describe the effects on templating accuracy when the ECM is moved along the anterior‐posterior axis from the centre of the hip. They determined that when the ECM is centred on the hip joint, an accurate cup size was achieved. A positioning error of 3.5 cm anterior or posterior to the hip joint, resulted in a full cup size of magnification error. We reviewed our results against the acetabular cup sizes of five major prosthesis vendors operating in Australia (See Table 5). From this, we calculated that there varied approximately 2–3 mm or greater templated difference between an OID of 8 and 12 cm. This is equal to at least one full cup size of magnification error and is comparable to the results of Ramme et al.^15^

**Table 5:** Examples of THA acetabular sizes across several different vendors used in Australia as per the 2016 National Joint Replacement Registry.^24^

Trident, Stryker® Cup Size (mm)^25^	Austin Moore, Altimed Cup Size (mm)^26^	Reflection, Smith&Nephew Cup Size (mm)^27^	Pinnacle®, DePuy Synthes Cup Size (mm)^28^	Versafitcup®, Medacta Cup Size (mm)^29^	Trilogy®, Zimmer® Cup Size (mm)^30^
40	38	46	48	46	44
42	40	48	50	48	46
44	42	50	52	50	48
46, 48	44	52	54	52	50
50, 52	46	54	56	54	52
54, 56	48	56	58	56	54
58, 60	50	58	60	58	56
62, 64	52	60	62	60	68
66, 68	54	62	64	62	60
70, 72	56	64	66	64	62
‐	58	66	68		64
‐	60	‐	70		66
‐	62	‐	72		68
‐	64	‐	‐		

**Figure 3:**
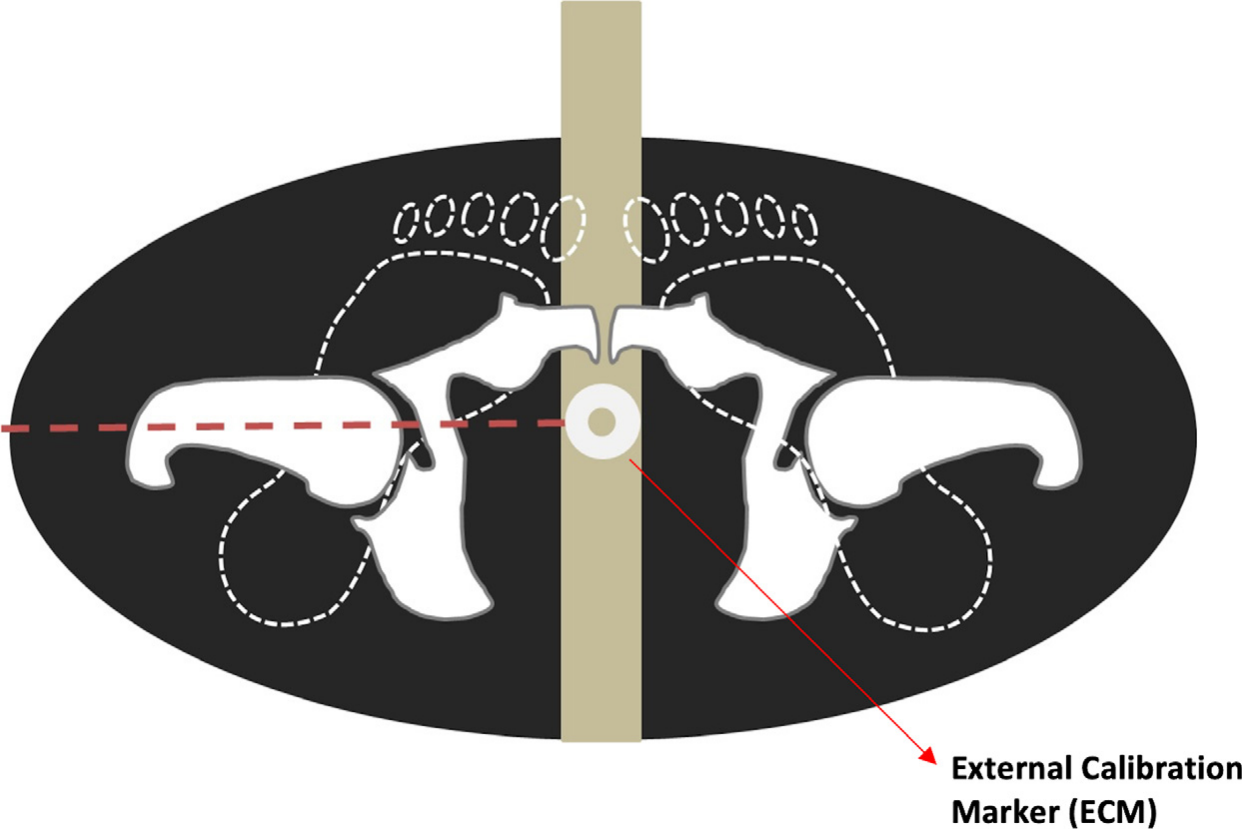
Correctly positioned external calibration marker (ECM) placed medially between a patient's internally rotated legs at the level of the greater trochanter equal to the level of the hip joint (Image courtesy of Olivia Ferraro).

This study also reiterates the importance of positioning the ECM as close to the level of the hip joint as possible (See Figure 3). Furthermore, the height should always be calculated with the patient’s legs internally rotated as this could potentially allow for a measured difference of up to 3–4 cm in height.

SIDs used within the literature are varied between 100 and 120 cm, for AP Pelvis imaging. The smallest recorded SID of 100 cm appears to be most commonly used.^5^, ^6^, ^11^, ^18^, ^20^, ^22^ As shown in our results the use of a SID of 100 cm concurred the greatest degree of magnification on the image and caused the greatest shift from the typical fixed MF value of 110% to 120%. Hornová et al.^12^ and Olmedo‐Garcia et al.^20^ suggested that standardised MF should be calibrated for each clinical site based upon individual protocols to account for this discrepancy in SID. Kwok et al.,^14^ calculated the degree of magnification in a series of images, ensuring that SID and SOD were included on the initial radiographs by the radiographer as part of their inclusion criteria. This allowed for an accurate MF to be determined for each radiograph. We believe that this method could be potentially time consuming in practice, especially in a busy hospital environment and also incite the potential for user dependent inaccuracies. Hence, it is our suggestion that the use of a correctly positioned ECM would concur similar degree of accuracy and eliminate the need for acquiring examination specific SID and SOD values.

It is important to note the influence of the emergency trolley for pelvic trauma imaging. This technique anecdotally observed in emergency X‐ray reduces patient transfer to the radiographic table in trauma imaging. To our knowledge, no other study has explored the effect this technique has on digital pre‐operative templating. As previously mentioned, the largest recorded MF for this study is 1.40 (140%) at 15 cm OID, when the X‐ray detector is placed under the emergency trolley at 100 cm SID. However as shown in Table 4, the smallest recorded MF value when the X‐ray detector is placed under the emergency trolley is 1.25 (125%) at 8 cm OID and 120 cm SID. This is clinically important as it suggests that an MF between 110% and 120%, as documented in the literature when not templating using an ECM, is clinically unachievable. This is due to the increased OID that is manifest in this technique. This is a clinically relevant finding and supports the need for radiographer compliance in appropriately positioning using an ECM. In our study, this method introduced an increase to the OID by 4 cm, described previously as raising a clinical impact of full acetabular cup size in discrepancy.

Another important imaging parameter explored in this study is the effect of lateral placement of an ECM. Boese et al. studied the influence of horizontal movement of objects within the projected beam on post‐op THA radiographs. They concluded that for most accurate templating results, the ECM must be placed medially between the patient’s legs in order to achieve equal magnification between the hip centre and the ECM. Our results support this (See Table 4), showing little deviation between the placement of the ECM laterally vs. medially with very minimal increase in MF when placed laterally.

It is important to consider potential limitations within our study. The study was intended to represent the measurement processes commonly used within clinical practice among radiographers using the line calliper tool. However, this introduces the potential for intra‐observer error. Calculation of the mathematical measurements using similar triangles was used to compare our results and suggest that they are accurate to less than 1.7 mm variation (See Figure S3 & Tables S1–S3). This mathematical method also helped support the importance of the use of an ECM, as there is a clear evidence of clinically important increased magnification with increasing distance from a point source of radiation. Further studies may look at reducing this intra‐observer error or may further investigate clinical outcomes attributed to inaccurate templating or rates of non‐compliance in templating.

## Conclusion

Digital pre‐operative templating for THA relies on accurate radiographic positioning and is dependent of the patient body habitus, radiographic parameters and imaging conditions selected by the radiographer. Based on the findings of this study, we believe that the use of a generalised MF does not account for deviation of these factors, and hence can be very inaccurate for varying SID, SOD, patient greater trochanter height (OID) values and imaging conditions. The use of appropriately positioned ECMs – placed medially between the patient’s internally rotated legs at the level of the greater trochanter, lowers the potential for magnification inaccuracies.

More emphasis should be placed in clinical practice on the importance of radiographer technique, to heighten accuracy and reproducibility when positioning an ECM for an AP pelvis X‐ray to lower the potential for inaccurate ECM placement. The authors suggest that standardisation of pelvic templating practice be achieved through implementation of a rigid pelvic imaging protocol and regular auditing across medical imaging departments.

## Conflict of Interest

The authors declare no conflict of interest.

## Supporting information

**Figure S1** X‐ray imaging of the trauma pelvis under the patient trolley, observed in emergency department X‐ray, as it reduces transfer of the patient to the radiographic table.
**Figure S2** Measuring system of the imaged size of the templating marker disc on a phantom pelvis of each radiograph using the line calliper tool.
**Figure S3** Method used to mathematically calculate the expected magnification using similar triangles.
**Table S1** Average disc measurements at 100, 110 and 120 cm SID values.
**Table S1a** Observer disc measurements and average disc measurement at 100 cm SID.
**Table S1b** Observer disc measurements and average disc measurement at 110 cm SID.
**Table S1c** Observer disc measurements and average disc measurement at 120 cm SID.
**Table S2** Average disc measurements at 100, 110 and 120 cm SID values.
**Table S2a** Observer disc measurements and average disc measurement at 100 cm SID.
**Table S2b** Observer disc measurements and average disc measurement at 110 cm SID.
**Table S2c** Observer disc measurements and average disc measurement at 120 cm SID.
**Table S3** Average disc measurements at 100, 110 and 120 cm SID values.
**Table S3a** Observer disc measurements and average disc measurement at 100 cm SID.
**Table S3b** Observer disc measurements and average disc measurement at 110 cm SID.
**Table S3c** Observer disc measurements and average disc measurement at 120 cm SID.
