# Supplementary material for: Pre‐operative templating for total hip arthroplasty: How does radiographic technique and calibration marker placement affect image magnification?
Source: J Med Radiat Sci. 2021 Feb 15;68(3):228–36. doi: 10.1002/jmrs.461 (PMC8424328; doi:10.1002/jmrs.461)
Supplement: Supplementary file 1 — Figure S1 X‐ray imaging of the trauma pelvis under the patient trolley, observed in emergency department X‐ray, as it reduces transfer of the patient to the radiographic table. Figure S2 Measuring system of the imaged size of the templating marker disc on a phantom pelvis of each radiograph using the line calliper tool. Figure S3 Method used to mathematically calculate the expected magnification using similar triangles. Table S1 Average disc measurements at 100, 110 and 120 cm SID values. Table S1a Observer disc measurements and average disc measurement at 100 cm SID. Table S1b Observer disc measurements and average disc measurement at 110 cm SID. Table S1c Observer disc measurements and average disc measurement at 120 cm SID. Table S2 Average disc measurements at 100, 110 and 120 cm SID values. Table S2a Observer disc measurements and average disc measurement at 100 cm SID. Table S2b Observer disc measurements and average disc measurement at 110 cm SID. Table S2c Observer disc measurements and average disc measurement at 120 cm SID. Table S3 Average disc measurements at 100, 110 and 120 cm SID values. Table S3a Observer disc measurements and average disc measurement at 100 cm SID. Table S3b Observer disc measurements and average disc measurement at 110 cm SID. Table S3c Observer disc measurements and average disc measurement at 120 cm SID. [file JMRS-68-228-s001.docx]

**Supporting Information**

Preoperative Templating for Total Hip Arthroplasty: How does Radiographic Technique and Calibration Marker Placement Affect Image Magnification?

Journal of Medical Radiation Sciences

Mia Holliday

Adam Steward

**Figure S1**: X-ray imaging of the trauma pelvis under the patient trolley, observed in emergency department x-ray, as it reduces transfer of the patient to the radiographic table. Image taken during 2020 Victorian Stage 4 COVID-19 restrictions.


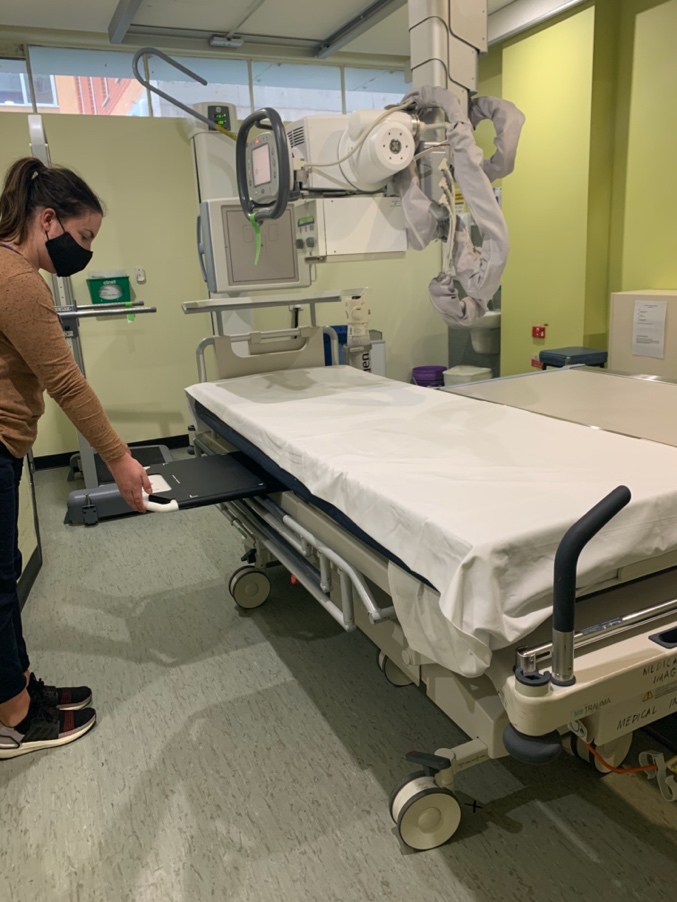


**Figure S2**: Measuring system of the imaged size of the templating marker disc on a phantom pelvis of each radiograph using the line calliper tool.

**
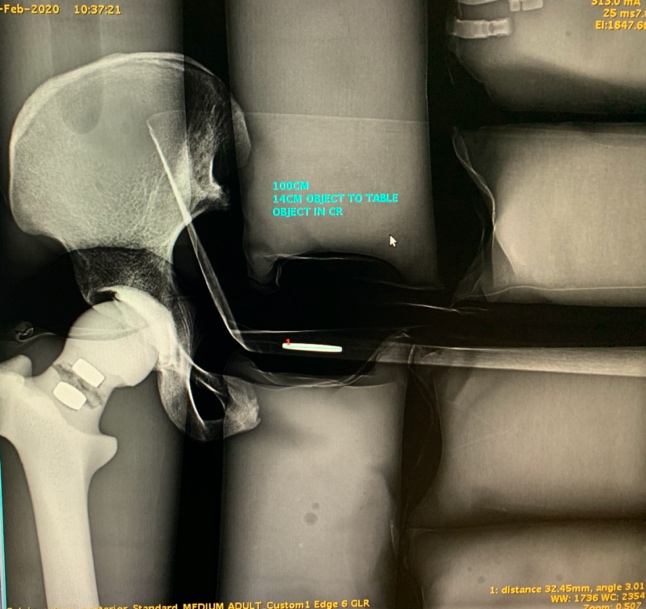
** **
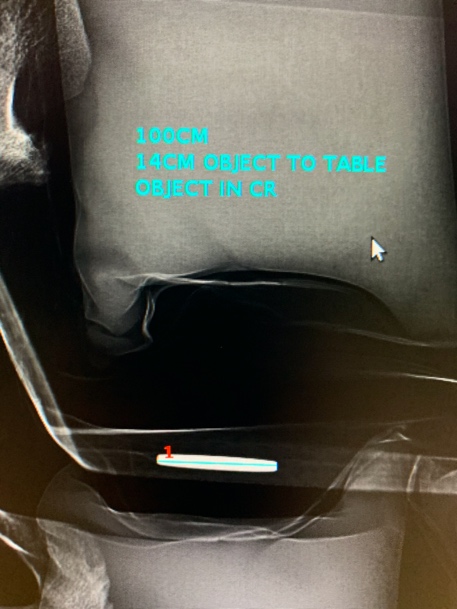
**

**Figure S3**: Method used to mathematically calculate the expected magnification using similar triangles.

**Radiation Point Source**


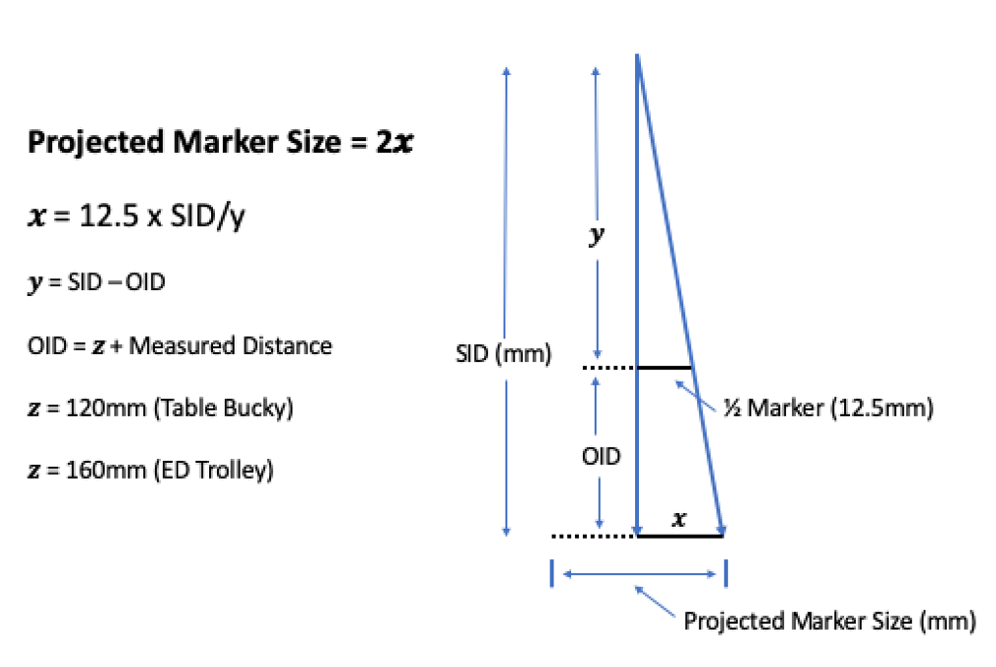


**Table S1**: Average disc measurements at 100, 110 and 120cm SID values. External Calibration Marker (ECM) placed with the central ray, x-ray detector placed in bucky underneath the x-ray table. †

| OID (cm) | ECM Measured Average **100cm SID** (mm) | MF **100cm SID** | ECM Measured Average **110cm SID** (mm) | MF **110cm SID** | ECM Measured Average **120cm SID** (mm) | MF **120cm SID** |
| --- | --- | --- | --- | --- | --- | --- |
| 8 | 29.7 | 1.19 | 29.6 | 1.18 | 28.9 | 1.16 |
| 9 | 30.0 | 1.20 | 29.8 | 1.19 | 29.3 | 1.17 |
| 10 | 30.3 | 1.21 | 30.1 | 1.20 | 29.6 | 1.19 |
| 11 | 31.1 | 1.24 | 30.5 | 1.22 | 29.8 | 1.19 |
| 12 | 31.6 | 1.26 | 30.7 | 1.23 | 30.2 | 1.21 |
| 13 | 31.7 | 1.27 | 31.1 | 1.24 | 30.4 | 1.22 |
| 14 | 32.3 | 1.29 | 31.6 | 1.27 | 30.4 | 1.22 |
| 15 | 32.9 | 1.31 | 31.9 | 1.28 | 31.0 | 1.24 |

† Abbreviations: object-to-image distance (OID), magnification factor (MF), source-to-image distance (SID)

**Table S1a**: Observer disc measurements and average disc measurement at 100cm SID. External Calibration Marker (ECM) placed with the central ray, x-ray detector placed in bucky underneath the x-ray table. †

| OID (cm) | ECM Measured Trial 1 (mm) | ECM Measured Trial 2 (mm) | ECM Measured Trial 3 (mm) | ECM Measured Average (mm) | Mathematical Measured (mm) | Difference to Measured Average (mm) | MF |
| --- | --- | --- | --- | --- | --- | --- | --- |
| 8 | 29.3 | 29.9 | 30.0 | 29.7 | 31.2 | 1.5 | 1.19 |
| 9 | 30.0 | 30.1 | 30.0 | 30.0 | 31.6 | 1.6 | 1.20 |
| 10 | 30.5 | 30.1 | 30.4 | 30.3 | 32.0 | 1.7 | 1.21 |
| 11 | 31.1 | 31.1 | 31.0 | 31.1 | 32.5 | 1.4 | 1.24 |
| 12 | 31.7 | 31.7 | 31.4 | 31.6 | 32.9 | 1.3 | 1.26 |
| 13 | 31.7 | 31.8 | 31.7 | 31.7 | 33.3 | 1.6 | 1.27 |
| 14 | 32.5 | 32.1 | 32.2 | 32.3 | 33.8 | 1.5 | 1.29 |
| 15 | 32.9 | 32.9 | 32.8 | 32.9 | 34.2 | 1.3 | 1.31 |

† Abbreviations: object-to-image distance (OID), magnification factor (MF)

**Table S1b**: Observer disc measurements and average disc measurement at 110cm SID. External Calibration Marker (ECM) placed with the central ray, x-ray detector placed in bucky underneath the x-ray table. †

| OID (cm) | ECM Measured Trial 1 (mm) | ECM Measured Trial 2 (mm) | ECM Measured Trial 3 (mm) | ECM Measured Average (mm) | Mathematical Measured (mm) | Difference to Measured Average (mm) | MF |
| --- | --- | --- | --- | --- | --- | --- | --- |
| 8 | 29.7 | 29.4 | 29.7 | 29.6 | 30.5 | 0.9 | 1.18 |
| 9 | 29.8 | 29.8 | 29.7 | 29.8 | 30.9 | 1.1 | 1.19 |
| 10 | 30.1 | 30.1 | 30.1 | 30.1 | 31.2 | 1.1 | 1.20 |
| 11 | 30.5 | 30.5 | 30.5 | 30.5 | 31.6 | 1.1 | 1.22 |
| 12 | 30.8 | 30.8 | 30.5 | 30.7 | 32.0 | 1.3 | 1.23 |
| 13 | 31.2 | 30.9 | 31.2 | 31.1 | 32.3 | 1.2 | 1.24 |
| 14 | 31.8 | 31.6 | 31.5 | 31.6 | 32.7 | 1.1 | 1.27 |
| 15 | 31.9 | 31.9 | 31.9 | 31.9 | 33.1 | 1.2 | 1.28 |

† Abbreviations: object-to-image distance (OID), magnification factor (MF)

**Table S1c**: Observer disc measurements and average disc measurement at 120cm SID. External Calibration Marker (ECM) placed with the central ray, x-ray detector placed in bucky underneath the x-ray table. †

| OID (cm) | ECM Measured Trial 1 (mm) | ECM Measured Trial 2 (mm) | ECM Measured Trial 3 (mm) | ECM Measured Average (mm) | Mathematical Measured (mm) | Difference to Measured Average (mm) | MF |
| --- | --- | --- | --- | --- | --- | --- | --- |
| 8 | 29.2 | 28.8 | 28.8 | 28.9 | 30.0 | 1.1 | 1.16 |
| 9 | 29.5 | 29.2 | 29.2 | 29.3 | 30.3 | 1.0 | 1.17 |
| 10 | 29.9 | 29.5 | 29.5 | 29.6 | 30.6 | 1.0 | 1.19 |
| 11 | 30.0 | 29.6 | 29.7 | 29.8 | 30.9 | 1.1 | 1.19 |
| 12 | 30.3 | 30.3 | 30.0 | 30.2 | 31.2 | 1.0 | 1.21 |
| 13 | 30.7 | 30.3 | 30.3 | 30.4 | 31.6 | 1.2 | 1.22 |
| 14 | 30.3 | 30.7 | 30.3 | 30.4 | 31.9 | 1.5 | 1.22 |
| 15 | 31.0 | 31.0 | 31.0 | 31.0 | 32.3 | 1.3 | 1.24 |

† Abbreviations: object-to-image distance (OID), magnification factor (MF)

**Table S2**: Average disc measurements at 100, 110 and 120cm SID values. External Calibration Marker (ECM) placed on the lateral edge of the detector, x-ray detector placed in bucky underneath the x-ray table. †

| OID (cm) | ECM Measured Average **100cm SID** (mm) | MF **100cm SID** | ECM Measured Average **110cm SID** (mm) | MF **110cm SID** | ECM Measured Average **120cm SID** (mm) | MF **120cm SID** |
| --- | --- | --- | --- | --- | --- | --- |
| 8 | 29.8 | 1.19 | 29.5 | 1.18 | 28.9 | 1.15 |
| 9 | 30.2 | 1.21 | 29.7 | 1.19 | 29.1 | 1.16 |
| 10 | 30.6 | 1.23 | 30.0 | 1.20 | 29.2 | 1.17 |
| 11 | 31.1 | 1.24 | 30.4 | 1.21 | 29.8 | 1.19 |
| 12 | 31.4 | 1.25 | 30.6 | 1.22 | 30.0 | 1.20 |
| 13 | 31.5 | 1.26 | 31.3 | 1.25 | 30.5 | 1.22 |
| 14 | 32.0 | 1.28 | 31.4 | 1.25 | 30.9 | 1.23 |
| 15 | 32.4 | 1.30 | 31.7 | 1.27 | 31.2 | 1.25 |

† Abbreviations: object-to-image distance (OID), magnification factor (MF), source-to-image distance (SID)

**Table S2a**: Observer disc measurements and average disc measurement at 100cm SID. External Calibration Marker (ECM) placed on the lateral edge of the detector, x-ray detector placed in bucky underneath the x-ray table. †

| OID (cm) | ECM Measured Trial 1 (mm) | ECM Measured Trial 2 (mm) | ECM Measured Trial 3 (mm) | ECM Measured Average (mm) | MF |
| --- | --- | --- | --- | --- | --- |
| 8 | 30.2 | 29.7 | 29.4 | 29.8 | 1.19 |
| 9 | 30.5 | 30.0 | 30.1 | 30.2 | 1.21 |
| 10 | 30.5 | 30.4 | 31.0 | 30.6 | 1.23 |
| 11 | 31.4 | 30.8 | 31.1 | 31.1 | 1.24 |
| 12 | 31.9 | 31.1 | 31.1 | 31.4 | 1.25 |
| 13 | 31.8 | 31.3 | 31.5 | 31.5 | 1.26 |
| 14 | 32.1 | 31.8 | 32.2 | 32.0 | 1.28 |
| 15 | 32.5 | 32.0 | 32.8 | 32.4 | 1.30 |

† Abbreviations: object-to-image distance (OID), magnification factor (MF)

**Table S2b**: Observer disc measurements and average disc measurement at 110cm SID. External Calibration Marker (ECM) placed on the lateral edge of the detector, x-ray detector placed in bucky underneath the x-ray table. †

| OID (cm) | ECM Measured Trial 1 (mm) | ECM Measured Trial 2 (mm) | ECM Measured Trial 3 (mm) | ECM Measured Average (mm) | MF |
| --- | --- | --- | --- | --- | --- |
| 8 | 29.9 | 29.4 | 29.2 | 29.5 | 1.18 |
| 9 | 30.1 | 29.4 | 29.7 | 29.7 | 1.19 |
| 10 | 30.3 | 29.8 | 30.0 | 30.0 | 1.20 |
| 11 | 30.7 | 30.1 | 30.3 | 30.4 | 1.21 |
| 12 | 31.1 | 30.4 | 30.3 | 30.6 | 1.22 |
| 13 | 31.6 | 31.3 | 31.1 | 31.3 | 1.25 |
| 14 | 31.5 | 31.2 | 31.4 | 31.4 | 1.25 |
| 15 | 32.0 | 31.5 | 31.6 | 31.7 | 1.27 |

† Abbreviations: object-to-image distance (OID), magnification factor (MF)

**Table S2c**: Observer disc measurements and average disc measurement at 120cm SID. External Calibration Marker (ECM) placed on the lateral edge of the detector, x-ray detector placed in bucky underneath the x-ray table. †

| OID (cm) | ECM Measured Trial 1 (mm) | ECM Measured Trial 2 (mm) | ECM Measured Trial 3 (mm) | ECM Measured Average (mm) | MF |
| --- | --- | --- | --- | --- | --- |
| 8 | 29.3 | 28.6 | 28.7 | 28.9 | 1.15 |
| 9 | 29.6 | 28.8 | 28.9 | 29.1 | 1.16 |
| 10 | 29.5 | 28.9 | 29.3 | 29.2 | 1.17 |
| 11 | 30.0 | 29.6 | 29.9 | 29.8 | 1.19 |
| 12 | 30.2 | 29.7 | 30.2 | 30.0 | 1.20 |
| 13 | 30.7 | 30.5 | 30.3 | 30.5 | 1.22 |
| 14 | 31.0 | 30.8 | 30.8 | 30.9 | 1.23 |
| 15 | 31.9 | 30.9 | 30.8 | 31.2 | 1.25 |

† Abbreviations: object-to-image distance (OID), magnification factor (MF)

**Table S3**: Average disc measurements at 100, 110 and 120cm SID values. External Calibration Marker (ECM) placed within the central ray, x-ray detector placed underneath the ED trolley. †

| OID (cm) | ECM Measured Average **100cm SID** (mm) | MF **100cm SID** | ECM Measured Average **110cm SID** (mm) | MF **110cm SID** | ECM Measured Average **120cm SID** (mm) | MF **120cm SID** |
| --- | --- | --- | --- | --- | --- | --- |
| 8 | 32.0 | 1.28 | 31.3 | 1.25 | 31.1 | 1.25 |
| 9 | 32.8 | 1.31 | 31.7 | 1.27 | 31.6 | 1.26 |
| 10 | 33.2 | 1.33 | 32.1 | 1.28 | 31.9 | 1.28 |
| 11 | 33.2 | 1.33 | 32.8 | 1.31 | 32.2 | 1.29 |
| 12 | 34.0 | 1.36 | 33.2 | 1.33 | 32.5 | 1.30 |
| 13 | 34.3 | 1.37 | 33.3 | 1.33 | 33.0 | 1.32 |
| 14 | 34.6 | 1.39 | 34.0 | 1.36 | 33.1 | 1.33 |
| 15 | 35.0 | 1.40 | 34.1 | 1.37 | 33.3 | 1.33 |

† Abbreviations: object-to-image distance (OID), magnification factor (MF), source-to-image distance (SID)

**Table S3a**: Observer disc measurements and average disc measurement at 100cm SID. External Calibration Marker (ECM) placed within the central ray, x-ray detector placed underneath the ED trolley. †

| OID (cm) | ECM Measured Trial 1 (mm) | ECM Measured Trial 2 (mm) | ECM Measured Trial 3 (mm) | ECM Measured Average (mm) | Mathematical Measured (mm) | Difference to Measured Average (mm) | MF |
| --- | --- | --- | --- | --- | --- | --- | --- |
| 8 | 32.0 | 32.0 | 32.0 | 32.0 | 32.9 | 0.9 | 1.28 |
| 9 | 32.8 | 32.8 | 32.8 | 32.8 | 33.3 | 0.5 | 1.31 |
| 10 | 33.2 | 33.2 | 33.2 | 33.2 | 33.8 | 0.6 | 1.33 |
| 11 | 33.2 | 33.2 | 33.2 | 33.2 | 34.2 | 1.0 | 1.33 |
| 12 | 34.0 | 34.0 | 34.0 | 34.0 | 34.7 | 0.7 | 1.36 |
| 13 | 34.4 | 34.4 | 34.0 | 34.3 | 35.3 | 0.9 | 1.37 |
| 14 | 34.8 | 34.9 | 34.2 | 34.6 | 35.7 | 1.1 | 1.39 |
| 15 | 35.1 | 35.1 | 34.7 | 35.0 | 36.2 | 1.2 | 1.40 |

† Abbreviations: object-to-image distance (OID), magnification factor (MF)

**Table S3b**: Observer disc measurements and average disc measurement at 110cm SID. External Calibration Marker (ECM) placed within the central ray, x-ray detector placed underneath the ED trolley. †

| OID (cm) | ECM Measured Trial 1 (mm) | ECM Measured Trial 2 (mm) | ECM Measured Trial 3 (mm) | ECM Measured Average (mm) | Mathematical Measured (mm) | Difference to Measured Average (mm) | MF |
| --- | --- | --- | --- | --- | --- | --- | --- |
| 8 | 31.2 | 31.2 | 31.6 | 31.3 | 32.0 | 0.7 | 1.25 |
| 9 | 31.7 | 31.7 | 31.7 | 31.7 | 32.4 | 0.7 | 1.27 |
| 10 | 32.1 | 32.1 | 32.2 | 32.1 | 32.7 | 0.6 | 1.28 |
| 11 | 32.8 | 32.8 | 32.8 | 32.8 | 33.1 | 0.3 | 1.31 |
| 12 | 33.1 | 33.2 | 33.2 | 33.2 | 33.5 | 0.3 | 1.33 |
| 13 | 33.3 | 33.3 | 33.3 | 33.3 | 34.0 | 0.7 | 1.33 |
| 14 | 34.0 | 34.0 | 34.1 | 34.0 | 34.4 | 0.4 | 1.36 |
| 15 | 34.0 | 34.0 | 34.4 | 34.1 | 34.8 | 0.7 | 1.37 |

† Abbreviations: object-to-image distance (OID), magnification factor (MF)

**Table S3c**: Observer disc measurements and average disc measurement at 120cm SID. External Calibration Marker (ECM) placed within the central ray, x-ray detector placed underneath the ED trolley. †

| OID (cm) | ECM Measured Trial 1 (mm) | ECM Measured Trial 2 (mm) | ECM Measured Trial 3 (mm) | ECM Measured Average (mm) | Mathematical Measured (mm) | Difference to Measured Average (mm) | MF |
| --- | --- | --- | --- | --- | --- | --- | --- |
| 8 | 31.0 | 31.0 | 31.4 | 31.1 | 31.2 | 0.1 | 1.25 |
| 9 | 31.8 | 31.1 | 31.8 | 31.6 | 31.6 | 0.0 | 1.26 |
| 10 | 31.7 | 32.0 | 32.1 | 31.9 | 31.9 | 0.0 | 1.28 |
| 11 | 32.1 | 32.1 | 32.5 | 32.2 | 32.3 | 0.1 | 1.29 |
| 12 | 32.4 | 32.1 | 32.9 | 32.5 | 32.6 | 0.1 | 1.30 |
| 13 | 32.9 | 32.9 | 33.3 | 33.0 | 33.0 | 0.0 | 1.32 |
| 14 | 33.2 | 32.9 | 33.3 | 33.1 | 33.3 | 0.2 | 1.33 |
| 15 | 33.3 | 33.0 | 33.7 | 33.3 | 33.7 | 0.4 | 1.33 |

† Abbreviations: object-to-image distance (OID), magnification factor (MF)
